# Disclosure of amyloid positron emission tomography results to individuals without dementia: a systematic review

**DOI:** 10.1186/s13195-018-0398-3

**Published:** 2018-07-28

**Authors:** Arno de Wilde, Marieke M. van Buchem, René H. J. Otten, Femke Bouwman, Andrew Stephens, Frederik Barkhof, Philip Scheltens, Wiesje M. van der Flier

**Affiliations:** 1 Department of Neurology & Alzheimer Center, Amsterdam Neuroscience, VU University Medical Center, Amsterdam, The Netherlands; 2 Medical Library, Vrije Universiteit Amsterdam, Amsterdam, the Netherlands; 3 Piramal Imaging GmbH, Berlin, Germany; 4 Department of Radiology & Nuclear Medicine, Amsterdam Neuroscience, VU University Medical Center, Amsterdam, the Netherlands; 5 Institutes of Neurology and Healthcare Engineering, UCL, London, UK; 6 Department of Epidemiology & Biostatistics, VU University Medical Center, Amsterdam, the Netherlands

**Keywords:** Amyloid PET, Disclosure, Non-demented, Psychological impact

## Abstract

**Background:**

Disclosure of amyloid positron emission tomography (PET) results to individuals without dementia has become standard practice in secondary prevention trials and also increasingly occurs in clinical practice. However, this is controversial given the current lack of understanding of the predictive value of a PET result at the individual level and absence of disease-modifying treatments. In this study, we systematically reviewed the literature on the disclosure of amyloid PET in cognitively normal (CN) individuals and patients with mild cognitive impairment (MCI) in both research and clinical settings.

**Methods:**

We performed a systematic literature search of four scientific databases. Two independent reviewers screened the identified records and selected relevant articles. Included articles presented either empirical data or theoretical data (i.e. arguments in favor or against amyloid status disclosure). Results from the theoretical data were aggregated and presented per theme.

**Results:**

Of the seventeen included studies, eleven reported empirical data and six provided theoretical arguments. There was a large variation in the design of the empirical studies, which were almost exclusively in the context of cognitively normal trial participants, comprising only two prospective cohort studies quantitatively assessing the psychological impact of PET result disclosure which showed a low risk of psychological harm after disclosure. Four studies showed that both professionals and cognitively normal individuals support amyloid PET result disclosure and underlined the need for clear disclosure protocols. From the articles presenting theoretical data, we identified 51 ‘pro’ and ‘contra’ arguments. Theoretical arguments in favor or against disclosure were quite consistent across population groups and settings. Arguments against disclosure focused on the principle of non-maleficence, whereas its psychological impact and predictive value is unknown. Important arguments in favor of amyloid disclosure are the patients right to know (patient autonomy) and that it enables early future decision making.

**Discussion:**

Before amyloid PET result disclosure in individuals without dementia in a research or clinical setting is ready for widespread application, more research is needed about its psychological impact, and its predictive value at an individual level. Finally, communication materials and strategies to support disclosure of amyloid PET results should be further developed and prospectively evaluated.

**Electronic supplementary material:**

The online version of this article (10.1186/s13195-018-0398-3) contains supplementary material, which is available to authorized users.

## Background

Amyloid-β aggregation in the brain is one of the neuropathological hallmarks of Alzheimer’s disease (AD) [1–3]. The introduction of 11-labeled Pittsburgh Compound B made it possible to detect amyloid-β deposition in vivo using positron emission tomography (PET) [4]. As part of the paradigm shift from clinical diagnoses to biomarker-supported diagnoses, amyloid PET has been incorporated as a biomarker in the diagnostic criteria for dementia and mild cognitive impairment (MCI) due to AD [5–9]. In addition, to stimulate studies assessing disease-modifying therapies at an early stage of disease, research criteria were introduced to define cognitively normal individuals with elevated cerebral amyloid-β as having preclinical AD [10, 11]. The advent of ^18^F-labeled amyloid PET ligands allowed widespread use for research purposes, while its subsequent regulatory agencies approval opened the way for use in clinical practice [12–15].

Given the lack of understanding of the predictive value of amyloid PET at an individual level [16–22], interpreting and communicating a PET result to amyloid-positive individuals who are not demented (yet) is challenging, and its impact has hardly been investigated [23]. Nevertheless, amyloid PET is already incorporated in anti-amyloid trials (> 15) as a screening instrument to identify individuals with Alzheimer’s pathology, either cognitively normal (CN) or with MCI, and result disclosure is a necessity of design [24, 25]. In addition, appropriate use criteria for clinical PET use indicate that patients with MCI could be considered for amyloid imaging to identify the underlying etiology, while CN individuals are considered inappropriate to scan given the limited prognostic value of PET [26, 27].

Taken together, amyloid PET result disclosure is increasingly being used in both research and clinical practice for different purposes, while evidence on its impact and safety is lagging behind. The aim of the current study was to systematically review the literature on the disclosure of amyloid PET in CN individuals and patients with MCI in both research and clinical settings.

## Methods

### Data search

This systematic review is based on the Preferred Reporting Items for Systematic Reviews and Meta-Analysis (PRISMA) statement [28]. We conducted a systemic literature search in four electronic databases on 28 March 2017: Embase, PubMed, the Cochrane library, and Web of Science. We developed the search strategy in collaboration with an information specialist from the VU Medical Center medical library. Search terms included controlled terms (MeSH in PubMed and Emtree in Embase) as well as free text terms. We used free text terms only in the Cochrane library. Search terms used were variations on the keywords ‘Alzheimer or dementia’, in combination with search terms comprising ‘disclosure’ and combined with search terms comprising ‘biomarker’ or ‘amyloid’. The full search strategies for all databases are presented in Additional file 1. The references of the included articles were further searched for relevant publications.

### Inclusion and exclusion criteria

We included articles if they reported on disclosure of amyloid PET results in patients with MCI and CN individuals. Given the limited number of studies with empirical data (e.g., randomized controlled trials, surveys, multiple case studies) we also considered reviews, perspectives, and point-of-views addressing theoretical arguments in favor or against amyloid PET disclosure. Articles had to be published in peer-reviewed journals, and all languages were accepted.

### Study selection

**Fig. 1:**
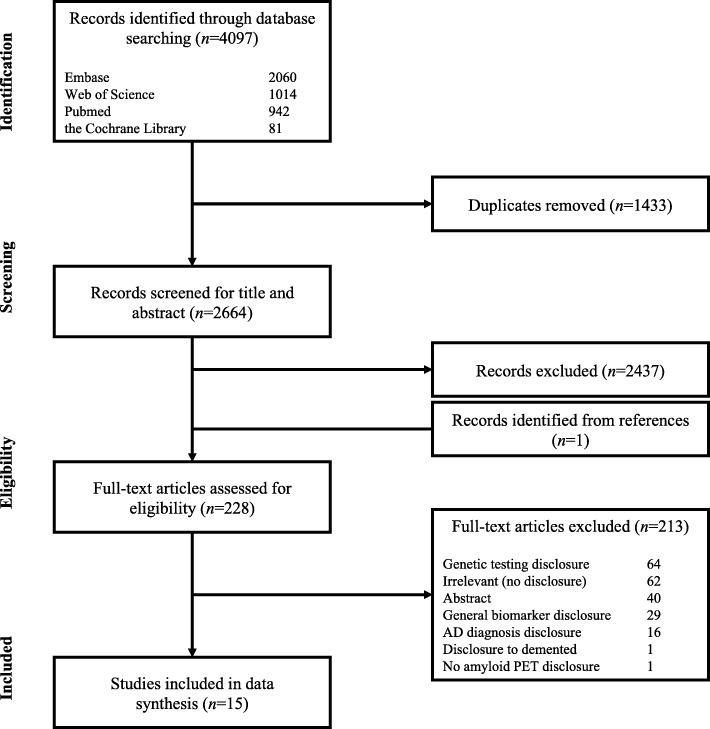
PRISMA flow diagram illustrating the process of study selection. AD Alzheimer’s disease

Our database search resulted in 2664 unique articles after removing duplicates. Titles and abstracts of all the identified articles were screened by two independent reviewers (AdW and MMvB). Screening for the title and abstract resulted in 228 articles for full-text assessment for eligibility. The same two reviewers assessed all full-text articles. In case of discrepancy, consensus was reached after discussion and consultation of a third reviewer (WMvdF). We included 15 articles for data extraction and data synthesis [5, 10, 29–41]. A flowchart that shows the results of the initial study selection is presented in Fig. 1. We additionally included two relevant articles that were published after we conducted our search [42, 43], resulting in a total of 17 articles.

### Data extraction and synthesis

Empirical data were extracted by two reviewers (AdW and MMvB), discussed, and checked by a third reviewer (WMvdF). We extracted data regarding main outcome measures and additional information on study design, population characteristics, methods, other relevant outcome measures, and the conclusions of the authors.

Arguments in favor or against disclosure in theoretical papers were identified and extracted by two reviewers (AdW and MMvB). In case of discrepancies between the reviewers, consensus was reached after discussion and consultation of a third reviewer (WMvdF). Due to a large variation in the content of the arguments, we composed a meta-summary. Finally, we grouped empirical data and arguments based on both the context (research versus clinic) and the population (CN versus MCI) for which the authors used them.

## Results

Of the fifteen included studies, nine reported empirical data on an aspect of amyloid PET result disclosure, while six provided theoretical arguments in favor or against disclosure.

### Empirical data in a research context

There was a large variation in the research questions and designs of the empirical studies, with two prospective cohort studies, three randomized controlled trials, three surveys, and a modified Delphi study. Table 1 shows an overview of the studies reporting empirical data.

**Table 1:** Overview of studies presenting empirical evidence

Author (year)	Objective	Setting	Design	Population (*n*)	Determinant	Assessment time points	Outcome measures	Results	Conclusion
Research context									
Researchers attitudes towards disclosure									
Shulman et al. (2013) [37]	To evaluate nondisclosure policy	Research	Survey	159 ADNI investigators and research staff	n.a.	n.a.	Practices and attitudes about returning amyloid imaging results	Small minority of CN (45%) and MCI (40%) participants do not request PET results. Most ADNI investigators did not return amyloid results to CN (94%) and MCI (90%). Majority would support disclosure to CN (58%) and MCI (73%) if FDA approval. Emphasized need for guidance on disclosure strategy	In view of FDA approval of amyloid imaging, disclosure to both CN and MCI is supported, and there is a need for 1) disclosure protocols and 2) knowledge on effect on participants
Cognitively normal study participants attitudes towards disclosure									
Ott et al. (2016) [36]	To assess the interest in knowledge of amyloid PET status	Research	Survey	164 CN	n.a.	n.a.	Wishes to learn amyloid PET status and, if so, reasons for wanting to know	81% desired to know amyloid status, motivated by desire to participate in AD research (73%), and to prepare family for illness (60%). Main reason for not desiring amyloid status was due to depressed feelings (40%). 12% indicated to use information for making plans on ending their life when memory loss becomes imminent	Stakeholders in AD prevention research generally wish to know information about their risk for developing AD to assist future planning.
Gooblar et al. (2015) [32]	To assess effect of education on disclosure attitude	Research	RCT	219 CN	Education intervention (education: *n* = 119, placebo: *n* = 110)	Pre- and postintervention	Interest in disclosure^a^	High preintervention interest (mean: 4.0 ± 1.1) in receiving amyloid imaging results, which significantly decreased after education versus placebo (OR 2.8, 95% CI 1.6–5.1) when controlled for preintervention level of interest	Learning about limitations of disclosure somewhat tempers interest
Grill et al. (2016) [33]	To assess the impact of amyloid disclosure on trial participation willingness	Research	RCT	132 self-reported CN older adults	Hypothetical ICF (transparent: *n* = 66, and blinded enrolment: *n* = 66)	Postintervention	Likelihood of enrollment^b^	No significant difference in willingness to participate between transparent (70%) and blinded (61%) group.	Requirement of amyloid disclosure may not slow recruitment to preclinical AD trials
Impact of disclosure									
Burns et al. (2017) [31]	To assess effect of amyloid disclosure	Research	Prospective cohort study	97 CN older adults	Amyloid status (amyloid positive: *n* = 27, amyloid negative: *n* = 70)	Before and at disclosure, and 6 weeks and 6 months post-disclosure	Depression (CES-D), anxiety (BAI), and test-related distress (IGT-AD)	No difference in depressive symptoms, slight increase in test-related distress, and group by time interaction in anxiety, without significant group differences	Disclosure has low risk of psychological harm
Lim et al. (2016) [35]	To assess impact of amyloid disclosure	Research	Prospective cohort study	11 CN older adults	Psychoeducational program (amyloid positive: *n* = 3, amyloid negative: *n* = 8)	Baseline, 9 and 18 months follow-up	Subjective complaints (MAC-Q), depression (DASS-D), anxiety (DASS-A), stress symptoms (DASS-S), and impact of events scale (IES-R)	Insufficient numbers for formal comparisons. Little change in psychological factors. Psychoeducational brochure rated as very useful. Disclosure of amyloid positivity motivated lifestyle changes	Disclosing amyloid status to CN older adults, who requested the information, seems safe
Vanderschaeghe et al. (2017) [42]	To assess how patients perceive and experience disclosure	Research	Semi-structured interviews	38 amnestic MCI patients	n.a.	2 weeks and 6 months post-disclosure	Fixed set of interview elements	2/8 PET-positive patients experienced emotional difficulties. 3/30 PET-negative patients doubted whether they received the correct result	Experienced advantages and disadvantages depended on outcome of PET result
Grill et al. (2017) [43]	To assess how patients perceive and experience disclosure	Clinic	Semi-structured telephone interview	26 patient-caregiver dyads, mostly with dementia, some MCI	n.a.	Post-disclosure (unspecified)	Fixed set of interview elements	Most patients would undergo amyloid PET again. Regardless of outcome, patients and caregivers commonly expressed relief on learning their results	Amyloid PET may provide information that patients and families find useful
Development of approach for amyloid disclosure									
Lingler et al. (2016) [30]	Development of amyloid disclosure protocol	Research	RCT and focus group	10 MCI care dyads	Mock disclosure (amyloid positive: *n* = 4, amyloid negative: *n* = 4, inconclusive: *n* = 2)	Postintervention	Satisfaction surveys, comprehension assessments, and focus groups	Recommendations included pretest counseling, screening for anxiety and depression, separate days for consent procedure, imaging, and disclosure, and follow-up to monitor the impact of disclosure, anxiety, and depression	MCI care dyads comprehended the information and were highly satisfied
Harkins et al. (2015) [29]	Development of amyloid disclosure protocol	Research	Modified Delphi method	Experts	n.a.	n.a.	n.a.	Recommendations included pretest counseling, the use of participants’ own brain images during disclosure, take-home materials, and follow-up to address emerging questions	Documents and process will be used in the A4 study
Clinical context									
Dementia specialists attitudes towards disclosure									
Klein and Kaye (2013) [34]	To assess attitudes of neurologists specializing in dementia towards the use of amyloid imaging	Clinic	Survey	135 neurologists specializing in dementia	n.a.	n.a.	Intention to use amyloid imaging in diagnosing AD and, if so, how they plan to use it	84% affirmed intention to use amyloid PET in practice. 24% intended to use PET for screening asymptomatic individuals. Patients should be counseled (92%)	Specialists generally support disclosure, but recognize complexity of scan interpretation, and need for patient counseling

*A4* Anti-Amyloid Treatment in Asymptomatic Alzheimer’s Disease, *AD* Alzheimer’s disease, *ADNI* Alzheimer’s Disease Neuroimaging Initiative, *BAI* Beck Anxiety Inventory, *CES-D* Center for Epidemiological Studies of Depression Scale, *CI* confidence interval, *CN* cognitively normal, *DASS-A* depression, anxiety, and stress scale, anxiety subscale, *DASS-D* depression, anxiety, and stress scale, depression subscale, *DASS-S* depression, anxiety, and stress scale, stress subscale, *FDA* Food and Drug Administration, *ICF* informed consent form, *IES-R* impact of events scale, revised edition, *IGT-AD* Impact of Genetic Testing for Alzheimer’s Disease, *MAC-Q* Memory Complaints Questionnaire, *MCI* mild cognitive impairment, *n.a.* non-applicable, *OR* odds ratio, *PET* positron emission tomography, *RCT* randomized controlled trial

^a^Five-point Likert scale

^b^Six-point Likert scale

### Researchers’ attitudes towards disclosure

Prior to the Food and Drug Administration (FDA) approval of ^18^F-florbetapir, Alzheimer’s Disease Neuroimaging Initiative (ADNI) investigators and research staff (*n* = 159) were interviewed about whether ADNI should change its policy of not returning amyloid imaging results [37]. Of interviewees with direct participant contact (*n* = 139), 45% reported that CN participants had never requested their PET results, and 40% reported this for participants with MCI. From the ADNI investigators, 94% reported that they “never” disclosed PET results to CN participants, while 90% did not return results to participants with MCI. A majority of respondents indicated that, upon FDA approval for ^18^F-florbetapir, they would support disclosing amyloid imaging results to MCI patients (73%) and participants with normal cognition (58%). Important reasons favoring disclosure (based on free-text answers) were based on the principles of ‘respect for autonomy’ and the ‘right to know’, and that it enables future planning and lifestyle changes, while concerns were expressed about potential psychological harms and impact on insurance. Respondents who endorsed disclosure stressed the need for developing standardized disclosure procedures, with the disclosure procedure needing to be rigorously studied with longitudinal outcomes to assess well-being.

### Cognitively normal study participants’ attitudes towards disclosure

Three studies assessed study participants’ interest in knowing their amyloid PET status, albeit using different research questions and study designs. One study surveyed the interest of CN participants (*n* = 164) in an Alzheimer’s prevention registry in knowing their amyloid status and their motivations [36], and 81% of participants expressed a wish to know their amyloid status, motivated by a desire to participate in AD research (73%) and to prepare their family for their illness (60%). Almost 12% indicated they would use the information to make plans for ending their life when memory loss becomes imminent. The main reason for not being interested in their amyloid status was because of the expectation of feeling depressed when amyloid was elevated (40%). The second study randomly assigned CN participants (*n* = 219) in a longitudinal aging study to an education intervention (*n* = 119) or placebo (*n* = 100) to measure pre- and postintervention interest in biomarker disclosure [32]. One of the items was interest in amyloid imaging results. The authors observed a high preintervention interest (mean 4.0 ± 1.1 on a five-point Likert scale, with 5 being extremely interested) in receiving amyloid PET results. After controlling for preintervention level of interest, an ordinal logistic regression showed that assignment (education versus placebo) significantly decreased interest (odds ratio (OR) 2.8, 95% confidence interval (CI) 1.6–5.1; *p* < 0.001), with the exemption of three subgroups: participants who 1) estimated their subjective AD risk greater than 50% (the mean), 2) reported having at least one parent with AD, and 3) attended few participant meetings. The third study assessed the likelihood of trial enrollment in older community volunteers with self-reported normal cognition (*n* = 132) after randomly assigning them to a hypothetical informed consent form where amyloid PET result disclosure was either present (transparent, *n* = 66) or absent (blinded, *n* = 66) [33]. There was no significant difference in the likeliness to enroll in clinical trials between the two groups (70% versus 61%).

### Impact of disclosure

Two studies assessed the impact of amyloid PET disclosure on depression, anxiety, and stress, while two studies performed semi-structured interviews to assess experiences after PET disclosure. The first study compared measures of depression, anxiety, and test-related distress at different time points between amyloid-positive (*n* = 27) and amyloid-negative (*n* = 70) trial participants [31]. Depressive symptoms were stable across visits and were not different between groups. For anxiety symptoms, there was a small increase immediately post-disclosure in the amyloid-positive group, which was not sustained. However, post-hoc analyses revealed no group differences at any time point. Amyloid-positive individuals had slightly higher levels of distress after PET result disclosure, and these were related to higher baseline levels of depression and anxiety. The second study assessed depression, anxiety, stress, impact of events, and subjective memory complaints in a small number of CN trial participants (*n* = 11) who explicitly requested their amyloid status and which they received after reading a psychoeducational brochure [35]. Despite an insufficient number to make formal comparisons, the impact of disclosure did not seem to be different between amyloid-positive (*n* = 3) and amyloid-negative (*n* = 8) participants. Participants considered the psychoeducational brochure to be very useful, and disclosure of amyloid positivity seemed to motivate lifestyle changes. The third study performed two semi-structured post-disclosure interviews with MCI patients (*n* = 38) as a substudy of a clinical trial in which participants could opt to know their PET result [42]. The results were assessed using qualitative content analysis. Most patients could recall the core message of their result disclosure in their own words. Two out of eight amyloid-positive patients experienced emotional difficulties (feeling worried, sadness) post-disclosure, whereas three of thirty amyloid-negative patients doubted whether they had received the correct result. Experienced advantages included the possibility of making practical arrangements. The fourth study performed semi-structured post-disclosure telephone interviews with 26 patient-caregiver dyads with whom a neurologist discussed the option of amyloid PET [43]. Clinical reasons for scanning varied but were generally considered consistent with the appropriate use criteria for amyloid imaging [26]. Most patients who chose to undergo amyloid imaging would opt for the scan again. Regardless of the PET result, patient and caregivers commonly expressed relief on learning the results. Some patients had expectations of the PET scan that are beyond its capabilities.

### Development of an approach for amyloid disclosure

Two studies reported on the development of amyloid PET disclosure materials in the context of clinical trials. The first study developed a process to maximize safety and effectiveness of disclosing amyloid imaging results to CN older adults participating in AD secondary preventions studies. They used a modified Delphi Method, consulting experts in the field of genetic testing and amyloid PET, to develop a consensus on best practices. Consensus was reached on the text for a brochure and a disclosure process. Recommendations included pretest counseling, screening for anxiety and depression, separate days for consent procedure, imaging, and disclosure, and follow-up to monitor the impact of disclosure, anxiety, and depression. The developed process and documents are currently used in the Anti-Amyloid Treatment in Asymptomatic Alzheimer’s Disease (A4) study [29]. The second study developed an approach for disclosure of amyloid imaging research results in patients with MCI [30]. They used simulated sessions during which MCI patients and their care dyads received fictitious but realistic information regarding their amyloid status, satisfaction surveys, comprehension assessments, and focus group data to evaluate the disclosure material. Recommendations included pretest counseling, the use of participants’ own brain images during disclosure, take-home materials, and follow-up to address emerging questions. The materials are currently used for trials at the University of Pittsburgh Alzheimer Disease Research Center.

### Empirical data in a clinical context

#### Dementia specialists’ attitudes towards disclosure

A cross-sectional survey among neurologists who specialized in dementia (*n* = 135) at US medical schools described their attitudes towards their intention to use amyloid imaging in clinical practice [34]. Of these, 84% affirmed their intention to use amyloid PET for evidence for (77%) or against (73%) a diagnosis of AD in cognitively impaired patients, while 24% intended to use amyloid PET for screening asymptomatic individuals. Most respondents (92%) felt that patients should be counseled in advance. A minority (16%) did not intend to use amyloid imaging, and they expressed concerns about costs, lack of improvement on existing diagnostic tools, and likelihood of misinterpretation of results by both patients and physicians.

##### Studies presenting theoretical arguments

From six articles, we identified a total of 51 arguments in favor (*n* = 22) or against (*n* = 29) amyloid PET result disclosure. We assessed which population (CN versus MCI) and context (research versus clinic) the authors used the arguments for. Subsequently, we grouped arguments into the following themes: ethical, social and legal, psychological and behavioral, and PET imaging characteristics (Table 2). Four studies focused their arguments on CN individuals, where their arguments where interchangeable (except for one) for a research or clinical context [10, 38, 40, 41]. Two studies provided arguments on individuals with MCI; one in a research and one in a clinical setting [5, 39]. There was considerable overlap between arguments regarding CN individuals or individuals with MCI, regardless of the context. For that reason, we decided to present arguments in favor or against disclosure grouped together.

**Table 2:** Overview of theoretical arguments in favor or against amyloid positron emission tomography (PET) result disclosure

		Cognitively normal		Mild cognitive impairment	
Category	Arguments	Research	Clinic	Research	Clinic
Pro					
Ethical	Patient autonomy	X^10,41^	X^10,41^	X^39^	X^5^
	Evidence of non-maleficence	X^10,40,41^	X^10,40,41^		
Social and legal	Cost and suffering reduction	X^38^	X^38^		
	Favors Alzheimer’s disease prevention	X^10^	X^10^		
Psychological and behavioral	Enables early decision making	X^10^,^38,41^	X^10,38,41^	X^39^	X^5^
	Clarifying effect of correct diagnosis	X^38^	X^38^	X^39^	X^5^
	Relief related to negative amyloid PET	X^38,41^	X^38,41^		
	Satisfies need for risk information	X^41^	X^41^		
PET imaging characteristics	Amyloid PET imaging is validated			X^39^	
	Clinical significance of amyloid PET				X^5^
Contra					
Ethical	Non-maleficence	X^10,38,41^	X^10,38,41^	X^39^	X^5^
	Lack of effective intervention	X10,^38^	X10,^38^	X^39^	
	Therapeutic misconception	X^2^	X^2^		
Social and legal	Unwanted personal implications	X^38,41^	X^10,38,41^		X^5^
	Social stigmatization	X^38,41^	X^38,41^		
Psychological and behavioral	Risk of psychological distress	X^10,38,41^	X^10,38,41^		X^5^
	Risk of false reassurance after negative PET	X^10,41^	X^10,41^	X^39^	
	Misinterpretation of positive amyloid PET	X^41^	X^41^		
PET imaging characteristics	Challenges related to inconclusive scans	X^10,38^	X^10,38^		X^5^
	Limited predictive value at level of individual	X^10,41^	X^10,41^		
	Variation on interpretation of PET results	X^10^	X^10^		

#### Arguments in favor of PET result disclosure

Frequently used arguments in favor of amyloid PET disclosure were based on the ethical principles of patient autonomy and non-maleficence (not to harm). Respect for patient autonomy is a foundation of medical ethics and is based on the notion that individuals should be able to make personal decisions based on their evaluation of the personal risks and benefits [10]. The argument of non-maleficence is mostly based on the parallel between amyloid PET disclosure and disclosure of apolipoprotein E (APOE) genotype, and the observation that in the latter case disclosure did not increase depression, anxiety, or stress [10, 40, 41]. In addition, amyloid PET result disclosure could potentially enable early decision making in terms of lifestyle changes (e.g., diet, exercise, cognitive training), trial participation, and planning for the future, while individuals still have full decision competence [5, 10, 38, 39, 41]. Also, a correct diagnosis using amyloid PET may be clarifying and appreciated by patients and their relatives, and add to a better understanding of their complaints and prognosis [5, 38, 39]. Notably, the argument that amyloid PET imaging is validated for use and that its clinical significance has been sufficiently established were only used in the context of result disclosure to individuals with MCI [5, 39].

#### Arguments against PET result disclosure

The most frequently used arguments against amyloid PET result disclosure were based on the ethical principle of avoiding potential harms, such as psychological distress (e.g., anxiety, depression, stress), or negative social-legal consequences (e.g., increased insurance premiums, the right to drive, maintain employability, or retain legal competence), especially in the absence of an effective disease-modifying treatment [5, 38, 41]. Another potential risk of disclosure is social stigmatization, whereby individuals can encounter discrimination in their social life or at the workplace [38, 41]. Finally, the predictive value of amyloid PET at an individual level is limited and warrants further research [10, 41].

## Discussion

Our systematic review of the literature on disclosure of amyloid PET results to individuals without dementia (cognitively normal and mild cognitive impairment) in both research and clinical contexts shows that the current body of empirical data is very weak. Theoretical arguments in favor or against disclosure were quite consistent across population groups and settings. Sparsely available data suggest that dementia specialists support disclosure in MCI patients clinically, while most researchers support disclosing amyloid PET results to individuals without dementia in research and they stress the importance of pretest counseling in this context. Cognitively normal individuals in a research setting are interested in learning about their amyloid status, and preliminary results show their risk of psychological harm seems low. There is a complete lack of studies in a clinical setting, however.

Three studies assessed the interests of cognitively normal individuals in learning about their amyloid PET results in a trial setting using very different designs [32, 33, 36]. Interest in disclosure of individual PET results was high in all studies, but somewhat tempered after learning the limitations of disclosure. One study reported that > 10% of patients indicated they would use the information of a positive PET to make plans for ending their life when memory loss becomes imminent [36]. A previous study on public perceptions of presymptomatic testing of AD reported a similar estimate, further emphasizing the need for psychological screening to identify individuals at a high risk of adverse psychological outcomes [44]. Within this context, requiring study partners for enrollment could be essential to ensure patient safety [45]. ADNI researchers were also in favor of disclosing amyloid PET results in a research setting, but stressed the need for pretest counseling and guidance on disclosure procedures [34, 37]. Of note, despite the fact that ADNI researchers were in favor of disclosing amyloid PET results, their preference has not been put into practice. In accordance, dementia specialists at US medical schools affirmed their intention to use amyloid PET in clinical practice for cognitively impaired individuals, while a small minority intent to use amyloid PET to screen asymptomatic individuals. These results illustrate that stakeholders are in agreement about amyloid results disclosure to CN and MCI individuals in a research setting while, in a clinical setting, this agreement is restricted to MCI individuals. Nevertheless, a recent study has demonstrated that amyloid PET has clinical impact in some individuals with subjective cognitive decline, while its usefulness in this population is currently also under investigation in the Amyloid Imaging to Prevent Alzheimer’s Disease (AMYPAD) study. Additionally, the importance of providing solid information on amyloid imaging and management of expectations with regard to the results is stressed [46–48].

Only two studies quantitatively assessed the effects of actual amyloid PET disclosure, both in cognitively normal individuals in a trial setting [31, 35]. Based on their results, it seems that disclosure of both positive and negative PET results has a low risk of psychological harm. Two studies that qualitatively assessed semi-structured interviews performed post-disclosure with either cognitively impaired study participants or their caregivers also reported few negative psychological outcomes, while some participants expressed a feeling of relief, even after a positive PET result [42, 43]. In addition, in an unselected memory clinic cohort, including patients with subjective cognitive decline (SCD) and MCI, subgroup analyses revealed unchanged measures of anxiety, and a decrease in uncertainty following amyloid PET [49]. However, it must be noted that the sample sizes of the studies were small, and most studies consisted of very select research populations. For example, participants were highly educated, and exclusion criteria included a history of neurological or psychiatric disorders, any significant systemic illness, or unstable medical condition. Given this common selection of very healthy and highly motivated research participants, the risk of adverse psychological events is probably reduced compared with an average clinical population. Thus far, quantitative data in a clinical setting are lacking completeness and the psychological effects of amyloid PET result disclosure could be very different in a patient population that attends a memory clinic. These patients will on average be less educated and have more physical and psychiatric comorbidity. Further studies are needed to assess the psychological risks in (clinical) populations.

Two studies developed a process to maximize the safety and effectiveness of amyloid PET result disclosure, and to guide the design and conduct of clinical trials that would require its disclosure (e.g., the A4 study) [29, 30]. The recommendations of both disclosure procedures, developed for CN individuals and individuals with MCI, were very similar. Recommendations included pretest counseling, pre- and post-disclosure monitoring of its psychological impact, and using separate days for counseling, imaging, and disclosure. A small but noticeable difference between the studies was the recommendation to use an individual’s own brain image for disclosure in the A4 study protocol. Recent findings from an A4 substudy on comprehension of positive PET results underline this recommendation; participants expressed a ‘desire for more specific, dimensional and quantitative information’, rather than a qualitative and dichotomous result [50].

Theoretical arguments in favor or against disclosure were quite consistent across population groups and settings. These similarities might demonstrate that it is not merely the arguments themselves that shape opinions on disclosure, but rather their varying ‘weight’ and ‘significance’ when used in different contexts. For individuals with MCI, there generally seems to be support for amyloid result disclosure, regardless of the setting, given that it is already part of research and clinical practice. However, the best approach to do so has yet to be determined. For CN individuals, on the contrary, there is a certain dichotomy [10]. Despite the lack of data on amyloid disclosure safety and effectiveness, and despite counter arguments, disclosure of amyloid status is common practice in the context of secondary AD prevention trials, while these same arguments are used to argue that disclosure is premature in a clinical setting.

Contrary to the paucity of data on amyloid PET disclosure, much more is known about the psychological and behavioral impact of disclosure of genetic biomarkers, such as the APOE e4 genotype [23]. In cognitively healthy individuals, disclosure of APOE e4 positivity is not associated with higher levels of anxiety and depression but it increases test-related distress. In addition, long-term care insurance uptake and health-related behavior changes increased, while it might affect subjective and objective memory functioning. However, it must be noted that research cohorts consisted almost exclusively of research participants without psychological complaints at baseline who had first-degree relatives with AD, limiting generalizability to other settings and groups. When comparing APOE e4 status disclosure with amyloid PET result disclosure, there are some major differences. First, carrying an APOE e4 allele is a risk factor for developing AD but, contrary to amyloid PET, does not reflect the presence of an ongoing pathophysiological process (i.e., the accumulation of brain amyloid beta). Second, APOE e4 studies almost exclusively assessed cognitively healthy research participants with a family history of AD, and these individuals may already suspect they are at increased risk for AD based on their positive family history.

Limitations of this systematic review are related to the limited body of literature on the topic of the effects of amyloid PET results disclosure. Studies were mainly focused on research settings and had widely different research questions and study designs. Only a few studies had quantitative outcome measures, and these were mainly focused on anxiety, depression, and stress. Given the potential impact of amyloid PET disclosure, it would also be interesting to assess its impact on employment, personal healthcare plans including lifestyle modification, and long-term planning. Available studies were mainly focused on amyloid disclosure in cognitively normal participants in the context of clinical trials, hampering translation of the results to clinical practice.

## Conclusions

This systematic review highlights the lack of data on amyloid PET result disclosure to individuals without dementia, especially in a clinical setting, and stresses the strong need for more studies in this context. This is critical for better understanding disclosure impact at a time when the use of amyloid PET is increasing in secondary AD prevention trials, and patients with SCD represent up to 25% of the clinical population [49]. The sparse data available suggest that disclosure of amyloid PET results has a low risk of psychological harm in the context of clinical trials, whereas both participants and professionals seem to support disclosure. Before amyloid PET result disclosure in individuals without dementia is ready for widespread application, more research is needed about its psychological impact, and its predictive value at an individual level. Finally, communication materials and strategies to support disclosure of amyloid PET results should be further developed and prospectively evaluated.

## Additional file

Additional file 1: Full search strategy per electronic database. (DOCX 38 kb)

## Abbreviations

A4: Anti-Amyloid Treatment in Asymptomatic Alzheimer’s Disease
AD: Alzheimer’s disease
ADNI: Alzheimer’s Disease Neuroimaging Initiative
AMYPAD: Amyloid Imaging to Prevent Alzheimer’s Disease
APOE: Apolipoprotein E
CI: Confidence interval
CN: Cognitively normal
FDA: Food and Drug Administration
MCI: Mild cognitive impairment
OR: Odds ratio
PET: Positron emission tomography
PRISMA: Preferred Reporting Items for Systematic Reviews and Meta-Analysis
SCD: Subjective cognitive decline
