# Supplementary material for: Disclosure of amyloid positron emission tomography results to individuals without dementia: a systematic review
Source: Alzheimers Res Ther. 2018 Jul 28;10:72. doi: 10.1186/s13195-018-0398-3 (PMC6064628; doi:10.1186/s13195-018-0398-3)
Supplement: Supplementary file 1 — Full search strategy per electronic database. (DOCX 38 kb) [file 13195_2018_398_MOESM1_ESM.docx]

**Additional file 1**

Search strategy in PubMed (28^th^ March 2017)

| **#** | **Query** | **Results** |
| --- | --- | --- |
| **#4** | #1 AND #2 AND #3 | **942** |
| **#3** | "Biomarkers"[Mesh] OR biomarker*[tiab] OR marker*[tiab] OR "Amyloid"[Mesh] OR "Amyloidosis"[Mesh] OR amyloid*[tiab] OR abeta*[tiab] OR a-beta*[tiab] OR risk*[tw] | **3251924** |
| **#2** | "Disclosure"[Mesh] OR disclos*[tiab] OR "Confidentiality"[Mesh] OR confidential*[tiab] OR inform[tiab] OR informing[tiab] OR "Ethics"[Mesh] OR "ethics" [Subheading] OR ethic*[tiab] | **360946** |
| **#1** | "Alzheimer Disease"[Mesh] OR alzheimer*[tiab] OR "Dementia"[Mesh:NoExp] | **156187** |

**Search strategy in Embase.com (28^th^ March 2017)**

| **#** | **Query** | **Results** |
| --- | --- | --- |
| **#4** | #1 AND #2 AND #3 | **2060** |
| **#3** | 'biological marker'/exp OR biomarker*:ti,ab OR marker*:ti,ab OR 'amyloid'/exp OR 'amyloidosis'/exp OR amyloid*:ti,ab OR abeta*:ti,ab OR a-beta*:ti,ab OR risk*:ti,ab,de | **3874090** |
| **#2** | disclos*:ti,ab OR 'confidentiality'/exp OR confidential*:ti,ab OR inform:ti,ab OR informing:ti,ab OR 'ethics'/exp OR ethic*:ti,ab | **465200** |
| **#1** | 'alzheimer disease'/exp OR 'dementia'/de OR alzheimer*:ti,ab | **247380** |

**Search strategy in the Cochrane Library (28^th^ March 2017)**

| **#** | **Query** | **Results** |
| --- | --- | --- |
| **#4** | #1 AND #2 AND #3 | **81** |
| **#3** | biomarker*:ti,ab,kw OR marker*:ti,ab,kw OR amyloid*:ti,ab,kw OR abeta*:ti,ab,kw OR “a-beta*”:ti,ab,kw OR risk*:ti,ab,kw | **1740278** |
| **#2** | disclos*:ti,ab,kw OR confidential*:ti,ab,kw OR inform:ti,ab,kw OR informing:ti,ab,kw OR ethic*:ti,ab,kw | **14446** |
| **#1** | alzheimer*:ti,ab,kw | **6606** |

**Search strategy in Web of Science (28^th^ March 2017)**

| **#** | **Query** | **Results** |
| --- | --- | --- |
| **#4** | #1 AND #2 AND #3 | **1014** |
| **#3** | TS=(biomarker* OR marker* OR amyloid* OR abeta* OR “a-beta*” OR risk*) | **2949459** |
| **#2** | TS=(disclos* OR confidential*:ti,ab OR inform OR informing OR ethic*) | **373278** |
| **#1** | TS=Alzheimer* | **191254** |
